# Supplementary material for: Cryo-EM structure of the bicarbonate receptor GPR30
Source: eLife. 2026 Aug 14;13:RP99874. doi: 10.7554/eLife.99874 (PMC13476069; doi:10.7554/eLife.99874)
Supplement: Supplementary file 4 [file elife-99874-fig1-figsupp1-data2.zip › Figure 1-figure supplement 1-source data 2/Figure 1-figure supplement 1-source data 2.pdf]

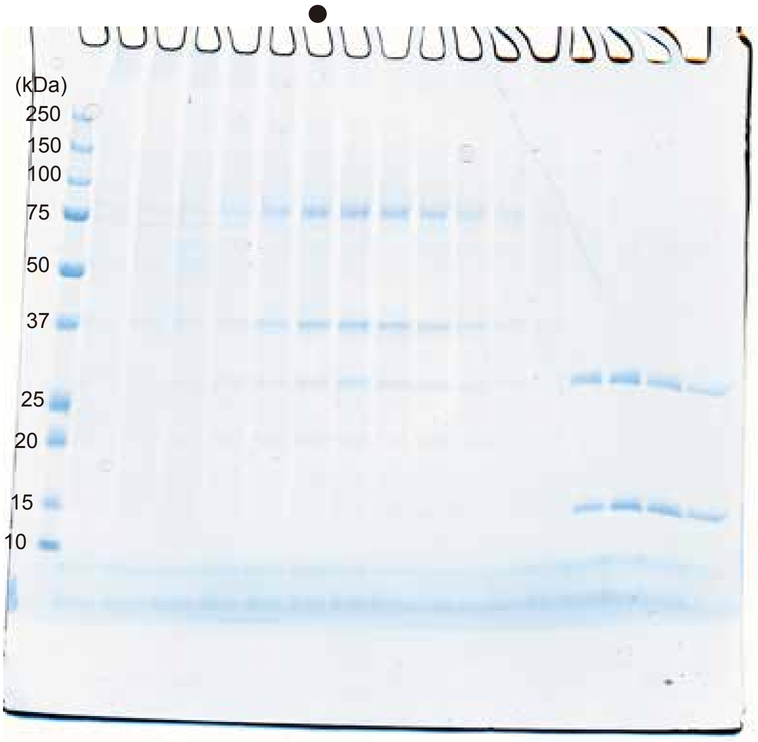

Figure 1.—figure supplement 1 source data 2.
Original SDS-PAGE data corresponding to Figure supplement 1, panel D. SDS-PAGE was performed on each fraction of interest from the size-exclusion chromatography. The marked lane was cropped as the representative image.
