# Supplementary material for: Cryo-EM structure of the bicarbonate receptor GPR30
Source: eLife. 2026 Aug 14;13:RP99874. doi: 10.7554/eLife.99874 (PMC13476069; doi:10.7554/eLife.99874)
Supplement: Supplementary file 6 [file elife-99874-fig3-figsupp4-data2.zip › Figure 3-figure supplement 4-source data 2/Figure 3-figure supplement 4-source data 2.pdf]

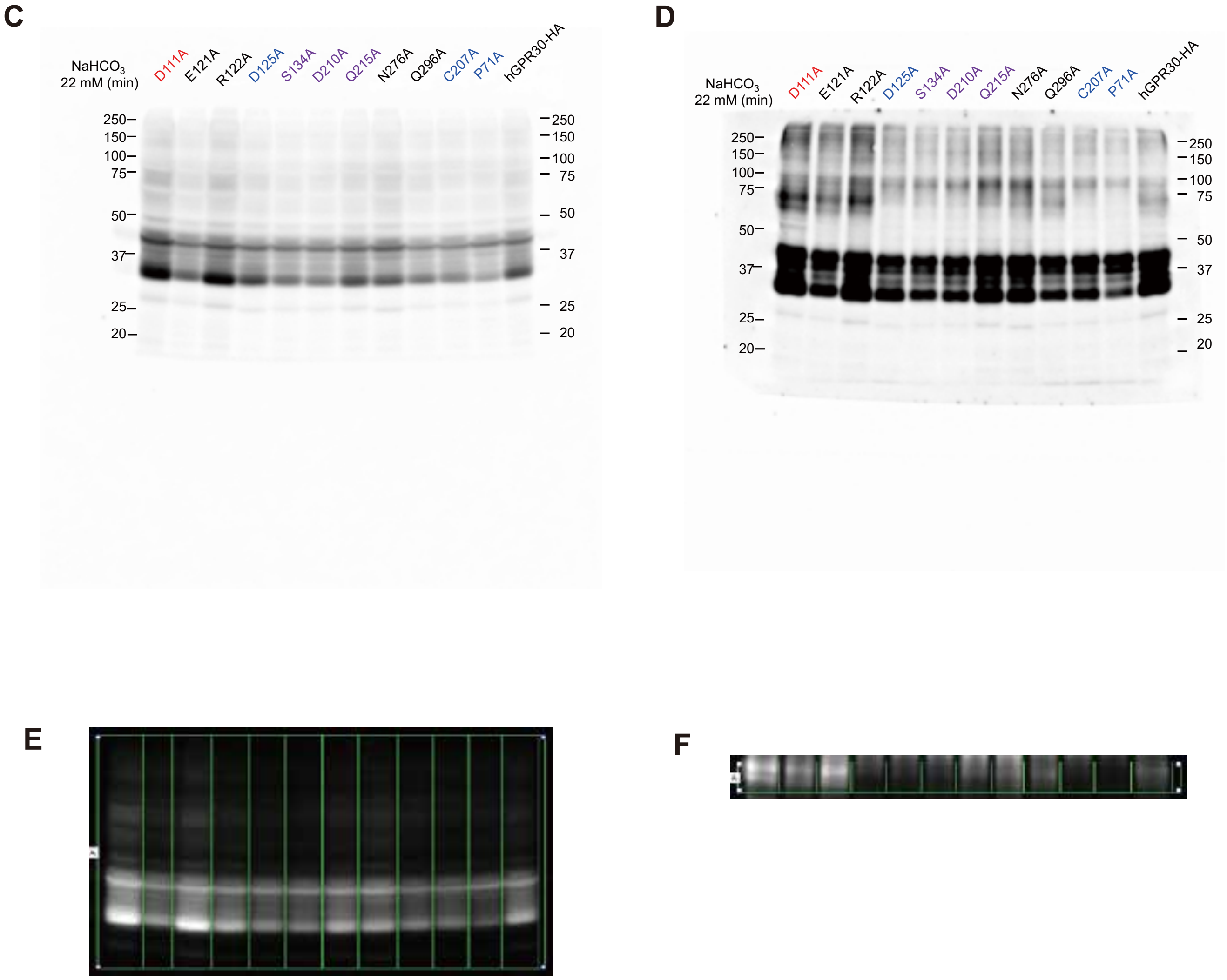

Figure 3.—figure supplement 4 source data 2.
Original Western blotting data corresponding to Figure supplement 4, panel C,D,E,F.
