# Supplementary material for: Cryo-EM structure of the bicarbonate receptor GPR30
Source: eLife. 2026 Aug 14;13:RP99874. doi: 10.7554/eLife.99874 (PMC13476069; doi:10.7554/eLife.99874)
Supplement: Supplementary file 6 [file elife-99874-fig3-figsupp4-data2.zip › Figure 3-figure supplement 4-source data 2/Figure 3-figure supplement 4D-source data 2.pdf]

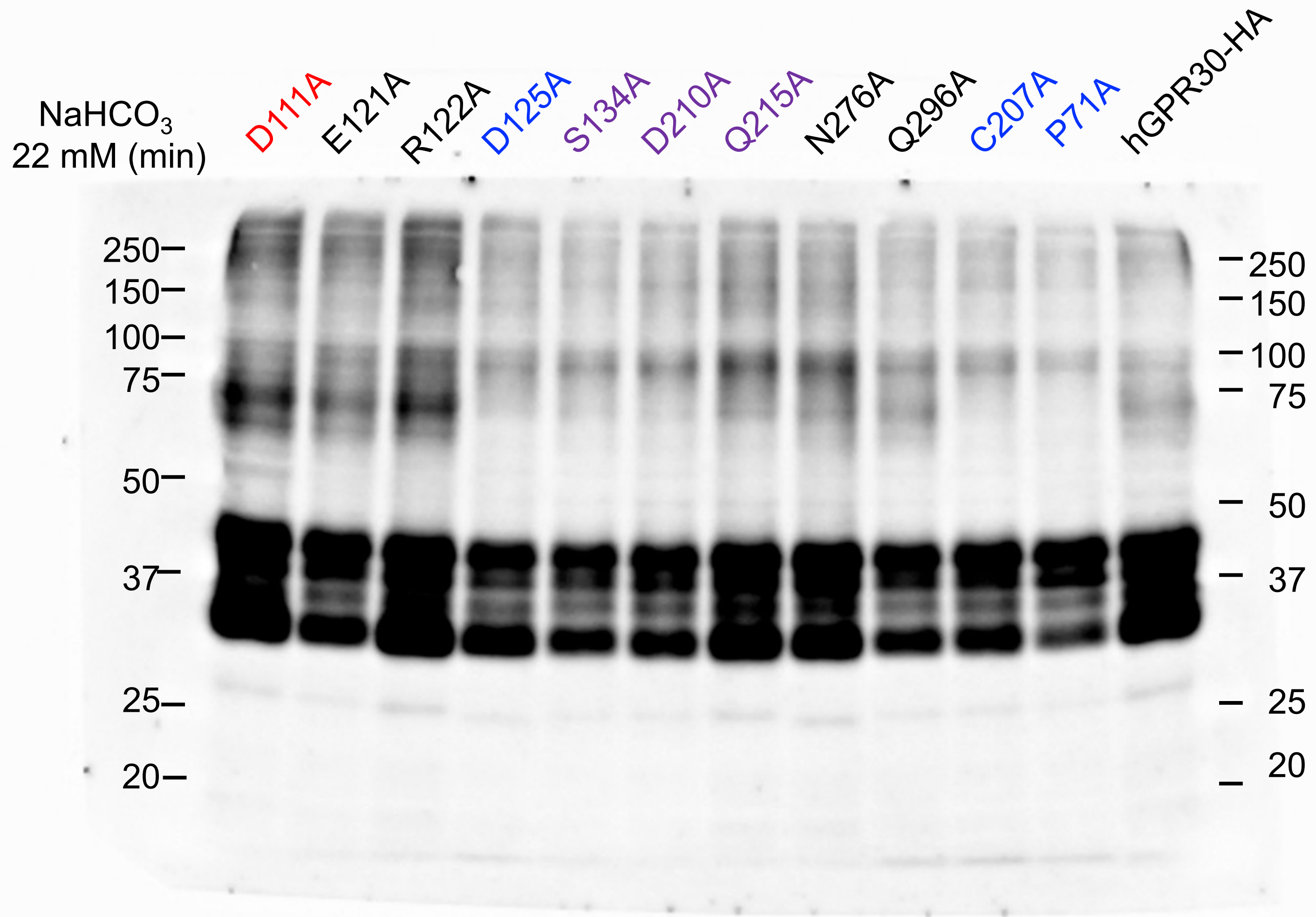
NaHCO$_3$
22 mM (min)
D111A
E121A
R122A
D125A
S134A
D210A
Q215A
N276A
Q296A
C207A
P71A
hGPR30-HA
250
150
100
75
50
37
25
20
250
150
100
75
50
37
25
20
