# Supplementary material for: Cryo-EM structure of the bicarbonate receptor GPR30
Source: eLife. 2026 Aug 14;13:RP99874. doi: 10.7554/eLife.99874 (PMC13476069; doi:10.7554/eLife.99874)
Supplement: Peer review history

# Editor report: eLife Assessment

**DOI:** 10.7554/eLife.99874.4.sa0

**Authors:** Randy B Stockbridge

**Affiliations:** University of Michigan United States

**Keywords:** Important, Solid

This study resolves a cryo-EM structure of the GPCR, human GPR30, which responds to bicarbonate and regulates cellular responses to pH and ion homeostasis. Understanding the ligand and the mechanism of activation is **important** to the field of receptor signaling and potentially facilitates drug development targeting this receptor. Structures and functional assays provide **solid** evidence for a potential bicarbonate binding site.

# Referee report: Reviewer #1 (Public review)

**DOI:** 10.7554/eLife.99874.4.sa1

[Editors' note: this version has been assessed by the Reviewing Editor without further input from the original reviewers.]

Summary:

This study resolves a cryo-EM structure of the GPCR, GPR30, in the presence of bicarbonate, which the author's lab recently identified as the physiological ligand. Understanding the ligand and the mechanism of activation is of fundamental importance to the field of receptor signaling. This solid study provides important insight into the overall structure and suggests a possible bicarbonate binding site.

Strengths:

The overall structure, and proposed mechanism of G-protein coupling are solid. Based on the structure, the authors identify a binding pocket that might accommodate bicarbonate. Although assignment of the binding pocket is speculative, extensive mutagenesis of residues in this pocket identifies several that are important to G-protein signaling. The structure shows some conformational differences with a previous structure of this protein determined in the absence of bicarbonate (PMC11217264). To my knowledge, bicarbonate is the only physiological ligand that has been identified for GPR30, making this study an important contribution to the field. However, the current study provides novel and important circumstantial evidence for the bicarbonate binding site based on mutagenesis and functional assays.

Weaknesses:

Bicarbonate is a challenging ligand for structural and biochemical studies, and because of experimental limitations, this study does not elucidate the exact binding site. Higher resolution structures would be required for structural identification of bicarbonate. The functional assay monitors activation of GPR30, and thus reports on not only bicarbonate binding, but also the integrity of the allosteric network that transduces the binding signal across the membrane. However, biochemical binding assays are challenging because the binding constant is weak, in the mM range.

The authors appropriately acknowledge the limitations of these experimental approaches, and they build a solid circumstantial case for the bicarbonate binding pocket based on extensive mutagenesis and functional analysis. However, the study does fall short of establishing the bicarbonate binding site.

# Referee report: Reviewer #2 (Public review)

**DOI:** 10.7554/eLife.99874.4.sa2

Summary:

In this manuscript, "Cryo-EM structure of the bicarbonate receptor GPR30," the authors aimed to enrich our understanding of the role of GPR30 in pH homeostasis by combining structural analysis with a receptor function assay. This work is a natural development and extension of their previous work on Nature Communications (PMID: 38413581). In the current body of work, they solved the cryo-EM structure of the human GPR30-G-protein (mini-Gsqi) complex in the presence of bicarbonate ions at 3.15 Å resolution. From the atomic model built based on this map, they observed the overall canonical architecture of class A GPCR and also identified 3 extracellular pockets created by ECLs (Pockets A-C). Based on the polarity, location, size, and charge of each pocket, the authors hypothesized that pocket A is a good candidate for the bicarbonate binding site. To identify the bicarbonate binding site, the authors performed an exhaustive mutant analysis of the hydrophilic residues in Pocket A and analyzed receptor reactivity via calcium assay. In addition, the human GPR30-G-protein complex model also enabled the authors to elucidate the G-protein coupling mechanism of this special class A GPCR, which plays a crucial role in pH homeostasis.

Strengths:

As a continuation of their recent Nature Communications publication, the authors used cryo-EM coupled with mutagenesis and functional studies to elucidate bicarbonate-GPR30 interaction. This work provided atomic-resolution structural observations for the receptor in complex with G-protein, allowing us to explore its mechanism of action, and will further facilitate drug development targeting GPR30. There were 3 extracellular pockets created by ECLs (Pockets A-C). The authors were able to filter out 2 of them and hypothesized that pocket A was a good candidate for the bicarbonate binding site based on the polarity, location, and charge of each pocket. From there, the authors identified the key residues on GPR30 for its interaction with the substrate, bicarbonate. Together with their previous work, they mapped out amino acids that are critical for receptor reactivity.

Weaknesses:

When we see a reduction of a GPCR-mediated downstream signaling, several factors could potentially contribute to this observation: (1) a reduced total expression of this receptor due to the mutation (transcription and translation issue); (2) a reduced surface expression of this receptor due to the mutation (trafficking issue); and (3) a dysfunctional receptor that doesn't signal due to the mutation.

Altogether, the wide range of surface expression across the different cell lines, combined with the different receptor function readouts, makes the cell functional data only partially support their structural observations.

# Referee report: Reviewer #3 (Public review)

**DOI:** 10.7554/eLife.99874.4.sa3

Summary

GPR30 responds to bicarbonate and plays a role in regulating cellular pH and ion homeostasis. However, the molecular basis of bicarbonate recognition by GPR30 remains unresolved. This study reports the cryo-EM structure of GPR30 bound to a chimeric mini-Gq in the presence of bicarbonate, revealing mechanistic insights into its G-protein coupling. Nonetheless, the study does not identify the bicarbonate-binding site within GPR30.

Strengths

The work provides strong structural evidence clarifying how GPR30 engages and couples with Gq.

Weaknesses

Several GPR30 mutants exhibited diminished responses to bicarbonate, but their expression levels were also reduced. As a result, the mechanism by which GPR30 recognizes bicarbonate remains uncertain.

# Author comment: Author response

**DOI:** 10.7554/eLife.99874.4.sa4

**Authors:** Shota Kaneda, Airi Jo-Watanabe, Hiroaki Akasaka, Hidetaka Oshima, Takehiko YOKOMIZO, Wataru Shihoya, Osamu Nureki

**Affiliations:** Department of Biological Sciences, The University of Tokyo Tokyo Japan; Juntendo University Graduate School of Medicine Tokyo Japan; Department of Biological Sciences, The University of Tokyo Tokyo Japan; Department of Biological Sciences, The University of Tokyo Tokyo Japan; Juntendo University Tokyo Japan; The University of Tokyo Bunkyo-ku Japan; Department of Biological Sciences, The University of Tokyo Tokyo Japan

The following is the authors’ response to the previous reviews

**Reviewer #1 (Public review):**

Summary:

This study resolves a cryo-EM structure of the GPCR, GPR30, in the presence of bicarbonate, which the author's lab recently identified as the physiological ligand. Understanding the ligand and the mechanism of activation is of fundamental importance to the field of receptor signaling. This solid study provides important insight into the overall structure and suggests a possible bicarbonate binding site.

Strengths:

The overall structure, and proposed mechanism of G-protein coupling are solid. Based on the structure, the authors identify a binding pocket that might accommodate bicarbonate. Although assignment of the binding pocket is speculative, extensive mutagenesis of residues in this pocket identifies several that are important to G-protein signaling. The structure shows some conformational differences with a previous structure of this protein determined in the absence of bicarbonate (PMC11217264). To my knowledge, bicarbonate is the only physiological ligand that has been identified for GPR30, making this study an important contribution to the field. However, the current study provides novel and important circumstantial evidence for the bicarbonate binding site based on mutagenesis and functional assays.

Weaknesses:

Bicarbonate is a challenging ligand for structural and biochemical studies, and because of experimental limitations, this study does not elucidate the exact binding site. Higher resolution structures would be required for structural identification of bicarbonate. The functional assay monitors activation of GPR30, and thus reports on not only bicarbonate binding, but also the integrity of the allosteric network that transduces the binding signal across the membrane. However, biochemical binding assays are challenging because the binding constant is weak, in the mM range.

The authors appropriately acknowledge the limitations of these experimental approaches, and they build a solid circumstantial case for the bicarbonate binding pocket based on extensive mutagenesis and functional analysis. However, the study does fall short of establishing the bicarbonate binding site.

We thank the reviewer for this thoughtful and constructive assessment of our revised manuscript. We are grateful for the recognition of the overall quality of the cryo-EM structure and the proposed mechanism of G-protein coupling, as well as for highlighting the importance of identifying bicarbonate as a physiological ligand for GPR30 and the contribution this work makes to the receptor signaling field. We also appreciate the reviewer’s careful and balanced discussion of the inherent challenges posed by bicarbonate as a low-affinity, small, negatively charged ligand, and we fully agree that, given current experimental limitations, our data provide circumstantial—rather than definitive—evidence for the binding site and that higher-resolution structures would be required for direct visualization. Importantly, we value the reviewer’s acknowledgement that we transparently describe these limitations and that our extensive mutagenesis and functional analyses nonetheless build a solid case for the proposed bicarbonate-binding pocket, which we believe will serve as a useful framework for future biochemical and structural investigation

**Reviewer #1 (Recommendations for the authors):**

Overall, the authors do a good job responding to the previous review, with updated structures and experimental data. I have two comments on the current version:

(1) When the authors compare their structure to a previously published structure of the same receptor, they say that the previous structure came out while the current manuscript was in revision (line 255). This is not correct. The previous manuscript was published May 14, 2024, and the current manuscript was received by eLife on May 20, 2024. This sentence should be corrected to "During the preparation of this manuscript..."

We corrected the sentence accordingly (line 259).

(2) Line 173: what other structures are the authors referring to? Citations should be included here.

Is Line 193 correct? We added citations (line 190).

**Reviewer #2 (Public review):**

Summary:

In this manuscript, "Cryo-EM structure of the bicarbonate receptor GPR30," the authors aimed to enrich our understanding of the role of GPR30 in pH homeostasis by combining structural analysis with a receptor function assay. This work is a natural development and extension of their previous work on Nature Communications (PMID: 38413581). In the current body of work, they solved the cryo-EM structure of the human GPR30-G-protein (mini-Gsqi) complex in the presence of bicarbonate ions at 3.15 Å resolution. From the atomic model built based on this map, they observed the overall canonical architecture of class A GPCR and also identified 3 extracellular pockets created by ECLs (Pockets A-C). Based on the polarity, location, size, and charge of each pocket, the authors hypothesized that pocket A is a good candidate for the bicarbonate binding site. To identify the bicarbonate binding site, the authors performed an exhaustive mutant analysis of the hydrophilic residues in Pocket A and analyzed receptor reactivity via calcium assay. In addition, the human GPR30-G-protein complex model also enabled the authors to elucidate the G-protein coupling mechanism of this special class A GPCR, which plays a crucial role in pH homeostasis.

Strengths:

As a continuation of their recent Nature Communications publication, the authors used cryo-EM coupled with mutagenesis and functional studies to elucidate bicarbonate-GPR30 interaction. This work provided atomic-resolution structural observations for the receptor in complex with G-protein, allowing us to explore its mechanism of action, and will further facilitate drug development targeting GPR30. There were 3 extracellular pockets created by ECLs (Pockets A-C). The authors were able to filter out 2 of them and hypothesized that pocket A was a good candidate for the bicarbonate binding site based on the polarity, location, and charge of each pocket. From there, the authors identified the key residues on GPR30 for its interaction with the substrate, bicarbonate. Together with their previous work, they mapped out amino acids that are critical for receptor reactivity.

Weaknesses:

When we see a reduction of a GPCR-mediated downstream signaling, several factors could potentially contribute to this observation: (1) a reduced total expression of this receptor due to the mutation (transcription and translation issue); (2) a reduced surface expression of this receptor due to the mutation (trafficking issue); and (3) a dysfunctional receptor that doesn't signal due to the mutation. In the current revision, based on the gating strategy, the surface expression of the HA-positive WT GPR30-expressing cells is only 10.6% of the total population, while the surface expression levels of the mutants range from 1.89% (P71A) to 64.4% (D111A). Combining this information with the functional readout in Figure 3F and G, as well as their previous work, the authors concluded that mutations at P71, E115, D125, Q138, C207, D210, and H307 would decrease bicarbonate responses. Among those sites,

E115, Q138, and H307 were from their previous Nature Comm paper.

Authors claim P71 and C207 make a structural-stability contribution, as their mutations result in a significant reduction in surface expression: P71A (1.89%) and C207A (2.71%). However, compared to 10.6% of the total population in the WT, (P71A is 17.8% of the WT, and C207A is 25.6% of the WT), this doesn't rule out the possibility that the mutated receptor is also dysfunctional: at 10 mM NaHCO3, RFU of WT is ~500, RFU of P71 and C207 are ~0.

The authors also interpret "The D125ECL1A mutant has lost its activity but is located on the surface" and only mention "D125 is unlikely to be a bicarbonate binding site, and the mutational effect could be explained due to the decreased surface expression". Again, compared to 10.6% of the total population in the WT, D125A (3.94%) is 37.2% of the WT. At 10 mM NaHCO3, the RFU of the WT is ~500, the RFU of D125 is ~0. This doesn't rule out the possibility that the mutated receptor is also dysfunctional. It is not clear why D125A didn't make it to the surface.

Other mutants that the authors didn't mention much in their text: D111A (64.4%, 607.5% of WT surface expression), E121A (50.4%, 475.5% of WT surface expression), R122 (41.0%, 386.8% of WT surface expression), N276A (38.9%, 367.0% of WT surface expression) and E218A (24.6%, 232.1% of WT surface expression) all have similar RFU as WT, although the surface expression is about 2-6 times more. On the other hand, Q215A (3.18%, 30% of WT surface expression) has similar RFU as WT, with only a third of the receptor on the surface.

Altogether, the wide range of surface expression across the different cell lines, combined with the different receptor function readouts, makes the cell functional data only partially support their structural observations.

We sincerely thank the reviewer for their careful reading and thoughtful evaluation of our manuscript on the cryo-EM structure of the bicarbonate receptor GPR30. We greatly appreciate the reviewer’s positive assessment of the overall significance of combining structural determination with extensive mutagenesis and functional assays to advance understanding of bicarbonate–GPR30 interactions and G-protein coupling, as well as their recognition that these atomic-level insights will be valuable for future mechanistic studies and drug-development efforts. We are also grateful for the reviewer’s constructive critique regarding the interpretation of reduced signaling in the context of variable surface expression across mutants, which highlights an important point about disentangling effects of expression/trafficking from intrinsic receptor dysfunction; these comments are highly insightful and will help us strengthen the clarity and rigor of our presentation and conclusions in the revised manuscript.

**Reviewer #2 (Recommendations for the authors):**

In this revision, the authors have made a significant effort to improve and validate the structural observations, as well as address the comments in the previous submission. They updated the functional assays and evaluated the receptor function by measuring intracellular calcium mobilization, which is a more direct measurement for the downstream signaling of hGPR30-Gq signaling. They also used flow cytometry with an HA-antibody for a more direct measurement of the surface expression of the receptor, replacing their previous assay that normalized to the housekeeping gene Na-K-ATPase.

I appreciate the effort the authors made to address the previous comments made by the reviewers. However, there are still some concerns about the current data.

(1) The authors have addressed my previous comment on untangling the mixture of their previous and new data in the "insights into bicarbonate binding" section. They have made it clear that the importance of E115, Q138, and H307 in the receptor-bicarbonate interaction was shown in their Nature Communications paper.

(2) The authors have addressed my previous comment on adding some content about the physiological concentration of HCO3, or referring more to their previous work about the rationale to select the bicarbonate dose in their functional assay.

(3) The authors have updated Figure 3

(4) The authors have updated Supplemental Figure 1 to show the full gel with molecular weight markers in the supplemental data to demonstrate the sample purity.

(5) The authors have updated the predicted model using AF3

(6) The authors added E218A as suggested before.

Some new suggestions for this R1:

(1) The wide range of surface expression across the different cell lines, combined with the different receptor function readouts, makes the cell functional data only partially support their structural observations.

We acknowledge this limitation. The wide range of surface expression among cell lines, together with differences in assay modalities, may introduce variability that complicates direct quantitative comparisons and therefore only partially supports the structural observations. Future work using more standardized expression systems and matched functional readouts will be important to strengthen the structure–function linkage.

(2) Line 101, "ICL1 and ECL1 contain short α helices", no α helix of ICL1 is shown in Figure 2C

We removed the word “ICL1” (line 98).

(3) For the unsolved region of ECL2, could the author put a dashed line connecting ECL2 with TM4? In the current Figure 2B, it looks like ECL2 connects TM3 and TM5.

According to the suggestion, we corrected Figure 2B.

(4) I appreciate that the authors updated the predicted model with AF3, but they didn't make it clear why they had the comparison between their cryo-EM structure (bicarbonate-activated G-protein-incorporated GPR30) and the predicted AF3 model (inactive GPR30)

We wish to assert the usefulness of experimental structures, not merely predictions. These include structures independent of receptor activation, such as SS bonds.

(5) I appreciate that the authors have addressed my previous comment on adding some content about the physiological concentration of HCO3, but it was still not clear to me why they picked 11 mM in Figure 3G for the bar graph. Also, since a dose-response curve was made in Figure 3F, why not just calculate and report the EC50 of NaHCO3 for each mutant?

Thank you for your comment. Thank you for the comment. We’ve calculated the EC50 of the calcium response and assessed its correlation with receptors’ cell surface expression. We chose 11 mM in Fig .3G since our previous paper in Nature Communications showed the EC50 value of IPs assay was around 11 mM. However, the calcium response was more sensitive and gave a lower value than expected. Therefore, according to your advice, we deleted the bar graph with 11 mM responses, calculated EC50, and drew pictures of the correlation among cell surface expression, EC50, and maximum responses (Figure 3F-I, Supplementary File 1). Moreover, we revised the explanation about this mutagenesis study (lines139-154 and 217-230).

(6) In the previous submission and comments, E218 was in close contact with bicarbonate in the previous Figure 4D (the bicarbonate is deleted in the new structure). I thank the authors for making an E218A mutant and performing the functional assay. As mentioned above, E218A (24.6%, 232.1% of WT surface expression) has a similar functional readout as WT. Doesn't this also indicate that E218A is partially broken, so you will need twice as much as WT to have the same downstream signal?

Thank you for your comment. In our revised manuscript, we described the correlation between cell surface expression and EC50 and found that cell surface expression and the response to bicarbonate are not correlated, which you mentioned in your review comment (Figure 3F-I, Supplementary File 1). There are many possibilities that could explain this: GPR30 localization in specific spots on the plasma membrane might limit the response stoichiometry, GPR30 might also work intracellularly to blunt the increased response because of more GPR30 expression on PM, redundant GPR30 on PM might be broken, or E118A might be less functional and need twice as much as WT. We will examine cell surface expression of GPR30 and its response to bicarbonate in a future study.

I would suggest that the authors in future studies consider using the Tet-on inducible cell lines, such as HEK293 Flp-In Trex. These cell lines will allow the authors to fine-tune the surface expression of their mutants to the same level with different doses of Tetracycline in their stable cell lines.

We appreciate your advice. We’ll introduce Tet-on inducible cell lines for future research.

**Reviewer #3 (Public review):**

Summary

GPR30 responds to bicarbonate and plays a role in regulating cellular pH and ion homeostasis. However, the molecular basis of bicarbonate recognition by GPR30 remains unresolved. This study reports the cryo-EM structure of GPR30 bound to a chimeric mini-Gq in the presence of bicarbonate, revealing mechanistic insights into its G-protein coupling. Nonetheless, the study does not identify the bicarbonate-binding site within GPR30.

Strengths

The work provides strong structural evidence clarifying how GPR30 engages and couples with Gq.

Weaknesses

Several GPR30 mutants exhibited diminished responses to bicarbonate, but their expression levels were also reduced. As a result, the mechanism by which GPR30 recognizes bicarbonate remains uncertain, leaving this aspect of the study incomplete.

We sincerely thank the reviewer for this thoughtful and balanced assessment of our manuscript, including the clear summary of the central advance and the constructive identification of remaining limitations. We particularly appreciate the recognition that our cryo-EM analysis provides strong structural evidence for how GPR30 engages and couples with Gq, and we agree that pinpointing the bicarbonate-binding site remains a critical open question. In the revised manuscript, we will make this point more explicit, clarify the interpretation of the mutagenesis results in light of reduced receptor expression for some variants, and further strengthen the presentation and discussion of what our current data do—and do not—allow us to conclude regarding bicarbonate recognition by GPR30

**Reviewer #3 (Recommendations for the authors):**

The authors have removed the bicarbonate assignment from their model and have addressed all of my concerns. In this study, or in future work, it would be advisable for the authors to explore the use of bicarbonate mimetics with higher binding affinity to facilitate more definitive structural characterization.

Thank you for this constructive suggestion. We agree that exploring bicarbonate mimetics with higher binding affinity would be an important next step to enable more definitive structural characterization of GPR30 and to strengthen mechanistic conclusions. In future work, we plan to pursue the identification and/or design of such mimetics, guided by the architecture and mutational landscape of the extracellular pocket described here, and to combine these ligands with optimized cryo-EM sample preparation and complementary functional assays to better stabilize and visualize the bound state.
